# Cytogenomic mapping and bioinformatic mining reveal interacting brain expressed genes for intellectual disability

**DOI:** 10.1186/1755-8166-7-4

**Published:** 2014-01-10

**Authors:** Fang Xu, Lun Li, Vincent P Schulz, Patrick G Gallagher, Bixia Xiang, Hongyu Zhao, Peining Li

**Affiliations:** 1 Department of Genetics, Yale University School of Medicine, New Haven, CT, USA; 2 Department of Epidemiology and Public Health, Yale University School of Medicine, New Haven, CT, USA; 3 Department of Pediatrics, Yale University School of Medicine, New Haven, CT, USA; 4 Hubei Bioinformatics and Molecular Imaging Key Laboratory, Huazhong University of Science and Technology, Wuhan, Hubei, P.R. China

**Keywords:** Intellectual disability, Critical regions, Brain expressed genes, Cross-region gene interrelation, Functional network

## Abstract

**Background:**

Microarray analysis has been used as the first-tier genetic testing to detect chromosomal imbalances and copy number variants (CNVs) for pediatric patients with intellectual and developmental disabilities (ID/DD). To further investigate the candidate genes and underlying dosage-sensitive mechanisms related to ID, cytogenomic mapping of critical regions and bioinformatic mining of candidate brain-expressed genes (BEGs) and their functional interactions were performed. Critical regions of chromosomal imbalances and pathogenic CNVs were mapped by subtracting known benign CNVs from the Databases of Genomic Variants (DGV) and extracting smallest overlap regions with cases from DatabasE of Chromosomal Imbalance and Phenotype in Humans using Ensembl Resources (DECIPHER). BEGs from these critical regions were revealed by functional annotation using Database for Annotation, Visualization, and Integrated Discovery (DAVID) and by tissue expression pattern from Uniprot. Cross-region interrelations and functional networks of the BEGs were analyzed using Gene Relationships Across Implicated Loci (GRAIL) and Ingenuity Pathway Analysis (IPA).

**Results:**

Of the 1,354 patients analyzed by oligonucleotide array comparative genomic hybridization (aCGH), pathogenic abnormalities were detected in 176 patients including genomic disorders in 66 patients (37.5%), subtelomeric rearrangements in 45 patients (25.6%), interstitial imbalances in 33 patients (18.8%), chromosomal structural rearrangements in 17 patients (9.7%) and aneuploidies in 15 patients (8.5%). Subtractive and extractive mapping defined 82 disjointed critical regions from the detected abnormalities. A total of 461 BEGs was generated from 73 disjointed critical regions. Enrichment of central nervous system specific genes in these regions was noted. The number of BEGs increased with the size of the regions. A list of 108 candidate BEGs with significant cross region interrelation was identified by GRAIL and five significant gene networks involving cell cycle, cell-to-cell signaling, cellular assembly, cell morphology, and gene expression regulations were denoted by IPA.

**Conclusions:**

These results characterized ID related cross-region interrelations and multiple networks of candidate BEGs from the detected genomic imbalances. Further experimental study of these BEGs and their interactions will lead to a better understanding of dosage-sensitive mechanisms and modifying effects of human mental development.

## Background

Intellectual disability (ID), also known as mental retardation, is characterized by significant limitations in intellectual functioning and adaptive behavior and is frequently associated with developmental delay (DD) and multiple congenital anomalies (MCA) [1]. Conventional cytogenetic evaluation of ID/DD/MCA showed an abnormality detection rate of 3.7% by karyotyping for large numerical and structural chromosomal abnormalities and up to 6.8% when combined with fluorescence in situ hybridization (FISH) analysis for targeted cryptic and subtelemeric rearrangements [2]. However, this molecular cytogenetic analysis was limited by the average Giemsa banding resolution of 5–10 megabase (Mb) and the number of targeted probes available in FISH analysis. For the past five years, genomic technologies using oligonucleotide array comparative genomic hybridization (aCGH) or a single nucleotide polymorphism (SNP) chip have been validated and recommended as first-tier genetic testing in replacing the conventional cytogenetic analysis [3,4]. Integration of genomic analysis into clinical cytogenetic services has filled the gap between the megabase-range G-band and the kilo-base level gene and proven to be highly effective in defining the genomic localizations and gene contents from recurrent genomic disorders, interstitial imbalances and subtelomeric rearrangements. The clinical application of aCGH for large case series of children and newborns has been reported; the diagnostic yield from cytogenomic analysis on pediatric patients with ID/DD/MCA and autism is about 12-20% [5-13].

It has been hypothesized that the general ID/DD phenotype from chromosomal segmental imbalances and genomic copy number variants (CNVs) is caused by dosage-sensitive mechanisms of involved genes. A chromosomal map which associated human brain congenital malformations with specific chromosomal regions had been constructed, but this low resolution map is of limited value for identifying dosage-sensitive genes [14]. Current cytogenomic analysis has facilitated accurate subtractive mapping of critical regions or intervals for identifying candidate genes in an increasing number of patients. For example, the breakpoints and gene content mediated by regional low copy number repeats (LCRs) of 15q12 for Prader-Willi or Angelman syndrome and of 22q11.2 for DiGeorge syndrome have been defined [15,16]. However, the lack of commonly accepted protocols for CNV prioritization and data concerning the gene dosage effect in the human genome makes it difficult to identify the disease-causing genes. To date it is still a technical challenge to predict candidate dosage-sensitive genes from the detected chromosomal and genomic abnormalities.

In the post-Genome era, many bioinformatic tools have been developed for biological interpretation of large gene lists derived from high-throughput experiments. Gene annotation enrichment analysis is one of the widely used approaches to statistically determine the most over-represented gene ontology (GO) terms and reveal the underlying biological processes in a group of genes [17]. Another strategy is to look for functionally related or interacting genes across genetic loci [18]. More recently, Gene Relationships Across Implicated Loci (GRAIL) has been applied to dissect common biological pathways affecting various phenotypes from SNP variants across genomic loci [19-21]. Similarly, Ingenuity Pathway Analysis (IPA), a web-delivered application, provides an integrated knowledge base comprising over 200,000 full text articles about the human genome and enables discovery and exploration of molecular interaction networks. It has been used to assess the functional biological networks and the candidate genes from the recurrent 16p11.2 microdeletions and the 1p34 microdeletion associated with autism [22,23].

In this study we used integrated cytogenomic mapping and bioinformatic mining to identify potential candidate genes and common gene interactions or networks from 176 genomic imbalances detected by aCGH from 1354 individuals with ID/DD/MCA and autism. Gene ontology enrichment of brain-expressed genes (BEGs) was noted by the gene functional classification analysis tool, Database for Annotation, Visualization, and Integrated Discovery (DAVID). Further gene-interaction analysis using GRAIL identified 108 candidate genes with related biological functions and strong interrelation cross imbalance regions. Pathway analysis using IPA denoted five significant gene networks involving cell cycle, cell-to-cell signaling, cellular assembly, cell morphology, and gene expression regulations. These findings provide insight into the cross-region interrelations of the BEGs and possibly the underlying dosage-sensitive mechanisms and modifying effects for human mental development.

## Results

### CNV characterization and classification

Of the 1354 pediatric patients referred for oligonucleotide aCGH analysis, 373 genomic imbalances were detected; 205 of these imbalances were denoted as pathogenic abnormalities in 176 patients (an individual may have more than one imbalance) and 168 imbalances were classified as VOUS (data not shown). The abnormality detection rate was 13.0% (176/1354). Excluding cases with simple aneuploidies, the 190 genomic abnormalities showed a mean genomic size of 6.6 Mb and a mean gene content of 47 genes per imbalance. The diagnosis yield of 14.0% by the 180 K microarray was slightly higher than the 12.5% by 44 K microarray. Approximately 60% of the genomic imbalances were deletions and 40% were duplications. Abnormalities detected at the terminal 10–15 Mb in large autosomes numbered 1 to 12 and terminal 5–10 Mb in small autosomes numbered 13 to 22 are usually considered to be subtelomeric rearrangements. Abnormalities found between centromere and subtelomeric, excluding the recurrent genomic disorders, are considered to be interstitial imbalances.

**Figure 1:**
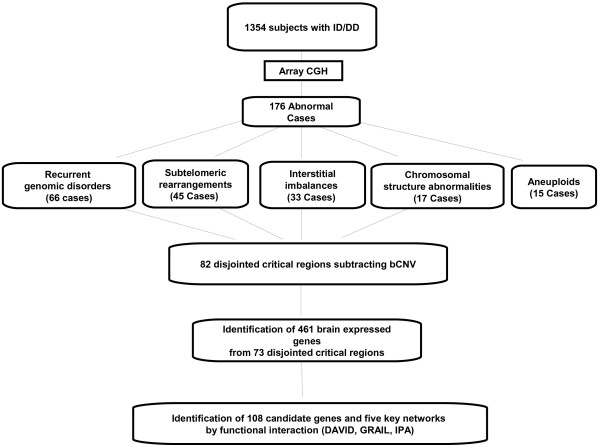
Flowchart for abnormality classification and bioinformatic filtration.

**Figure 2:**
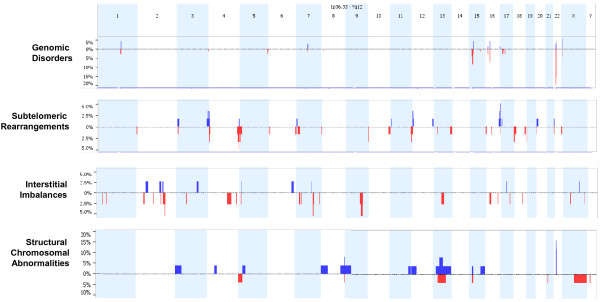
**A genome-wide view of detected abnormalities.** Detected recurrent genomic disorders, subtelomeric rearrangements, interstitial imbalances, and structural chromosomal abnormalities are shown in its category with genomic location and relative frequency (downward red bar for deletion and upward blue bar for duplication).

**Figure 3:**
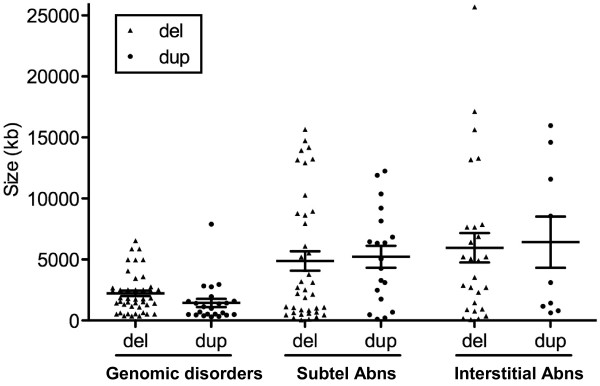
**The size distribution of genomic disorders, subtelomeric rearrangements and interstitial imbalances.** The average sizes for deletions and duplications are 2.2 Mb and 1.4 Mb for genomic disorders, 4.9 Mb and 5.2 Mb for subtelomeric abnormalities (abns), and 6.0 Mb and 6.2 Mb interstitial abnormalities, respectively.

The workflow of cytogenomic mapping and bioinfomatic mining is outlined in Figure 1. Details of the aCGH results are summarized in Additional file 1: Table S1, Additional file 2: Table S2, Additional file 3: Table S3, Additional file 4: Table S4. The detected abnormalities were classified into five categories: recurrent genomic disorders mediated by regional low-copy repeats in 66 cases (37.5%, 66/176) (Additional file 1: Table S1), subtelomeric abnormalities resulting from terminal deletions or unbalanced rearrangements in 45 cases (25.6%) (Additional file 2: Table S2), sporadic interstitial deletions and duplications in 33 cases (18.8%) (Additional file 3: Table S3), chromosomal structure abnormalities in 17 cases (9.7%) (Additional file 4: Table S4) and simple aneuploidies in 15 cases (8.5%). A genome-wide view of the detected genomic disorders, subtelomeric rearrangements, interstitial imbalances and chromosomal structural abnormalities is shown in Figure 2. The most frequently detected genomic disorders were deletions of the 22q11.21 in 13 cases (OMIM188400) and the reciprocal duplication (OMIM 608363) in three cases. The subtelomeric rearrangements included simple terminal deletions and duplications identified in 31 cases and de novo or familial subtelomeric rearrangements in 14 cases. The size distribution of deletions and duplications from genomic disorders, subtelomeric and interstitial rearrangements is shown in Figure 3. The average size of deletions for subtelomeric and interstitial rearrangements was 4.9 Mb and 6.0 Mb while the average size of duplications was 5.2 Mb and 6.4 Mb, respectively. The 15 cases of simple aneuploidies included seven cases of trisomy 21, one trisomy 13 and seven cases of sex chromosome aneuploidies.

### Functional annotation enrichment

Excluding the 15 cases with simple aneuploidies, cytogenomic mapping was performed on the remaining 161 cases. After subtracting the bCNV, fine-mapping the critical regions and combining overlapping regions, 82 disjointed critical regions belonging to recurrent genomic disorders, subtelomeric and interstitial regions were defined; a total of 4080 genes were retrieved from the UCSC RefGene table (Additional file 5: Table S5). Functional annotation tool DAVID was used to analyze each gene list from recurrent genomic disorders, subtelomeric rearrangements, interstitial imbalances and structural chromosomal abnormalities with gene tissue expression annotations derived from uniprot (UP_TISSUE). Gene lists in the genomic disorder, subtelomeric and interstitial critical regions were noted to be enriched for brain specific genes (p < 0.05). To avoid biased tissue information from Uniprot and to understand the expression pattern comprehensively, the tissue expression enrichment was further investigated using GNF_U133A, a world-class tissue expression database. The results showed that genes in these four lists are all enriched in central nervous systems (CNS) (Additional file 6: Figure S1). Genes in recurrent genomic disorders and subtelomeric regions are preferentially expressed in medulla oblongata (p < 0.005), hypothalamus (p < 0.05) and pituitary (p < 0.1), while genes in interstitial and structural abnormal regions are enriched in cingulate cortex (p < 0.05), cerebellum (p < 0.1) and occipital lobe (p < 0.1). The enrichments were confirmed by 1000 runs of permutation. This observation indicated that there is brain functional gene enrichment in the defined critical regions.

### Relationship between the numbers of the BEGs and the size of the abnormal loci

Based on findings that genes preferentially expressed in variable sites of the CNS are enriched in the critical regions, candidate BEGs were sorted out using the method described by Raychaudhuri, which examined and compared gene expression pattern in different tissues with the expression pattern in brain or spinal cord [18]. Genes with p < 0.01 were identified as preferentially expressed in the CNS system. A total of 461 BEGs from 73 disjointed critical regions in 133 cases were defined (Additional file 7: Table S6). In nine disjointed critical regions, there were no BEGs using this approach.

**Figure 4:**
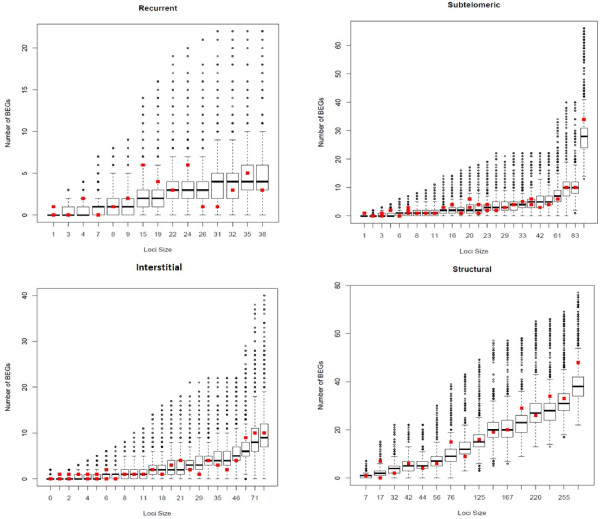
**Boxplots showing the permutation-based empirical distributions of the number of BEGs observed in the critical regions of different gene content sizes (measured by gene numbers).** For each of the 82 targeted abnormal regions, a set of 10,000 random genomic loci with gene content size defined by the same number of genes as the target regions was drawn from the genome. The X-axis represents the number of genes located in each regions, and the Y-axis stands for the numbers of BEGs observed. The actual numbers of BEGs detected in each abnormal region was shown as big solid red square.

To evaluate the distribution of the number of BEGs in the critical regions and test whether the number of BEGs was over-represented in comparison to random loci with the same number of genes, permutation-based empirical distributions were conducted for the 16 regions in recurrent genomic disorders, 39 disjointed regions in subtelomeric rearrangements, 34 disjointed regions in interstitial imbalances and 17 disjointed regions in structural abnormalities. For each abnormal region, the number of BEGs in the random loci was determined and compared with that of the abnormal regions by boxplot. Although variations were noted in some recurrent genomic disorder regions, the overall distribution showed that the number of BEGs in detected abnormal regions is positively correlated to the size of the region (Figure 4). This observation indicated that the number of BEGs likely increases with the size of gene content of the imbalance regions.

### Identification of candidate genes and functional interaction

**Figure 5:**
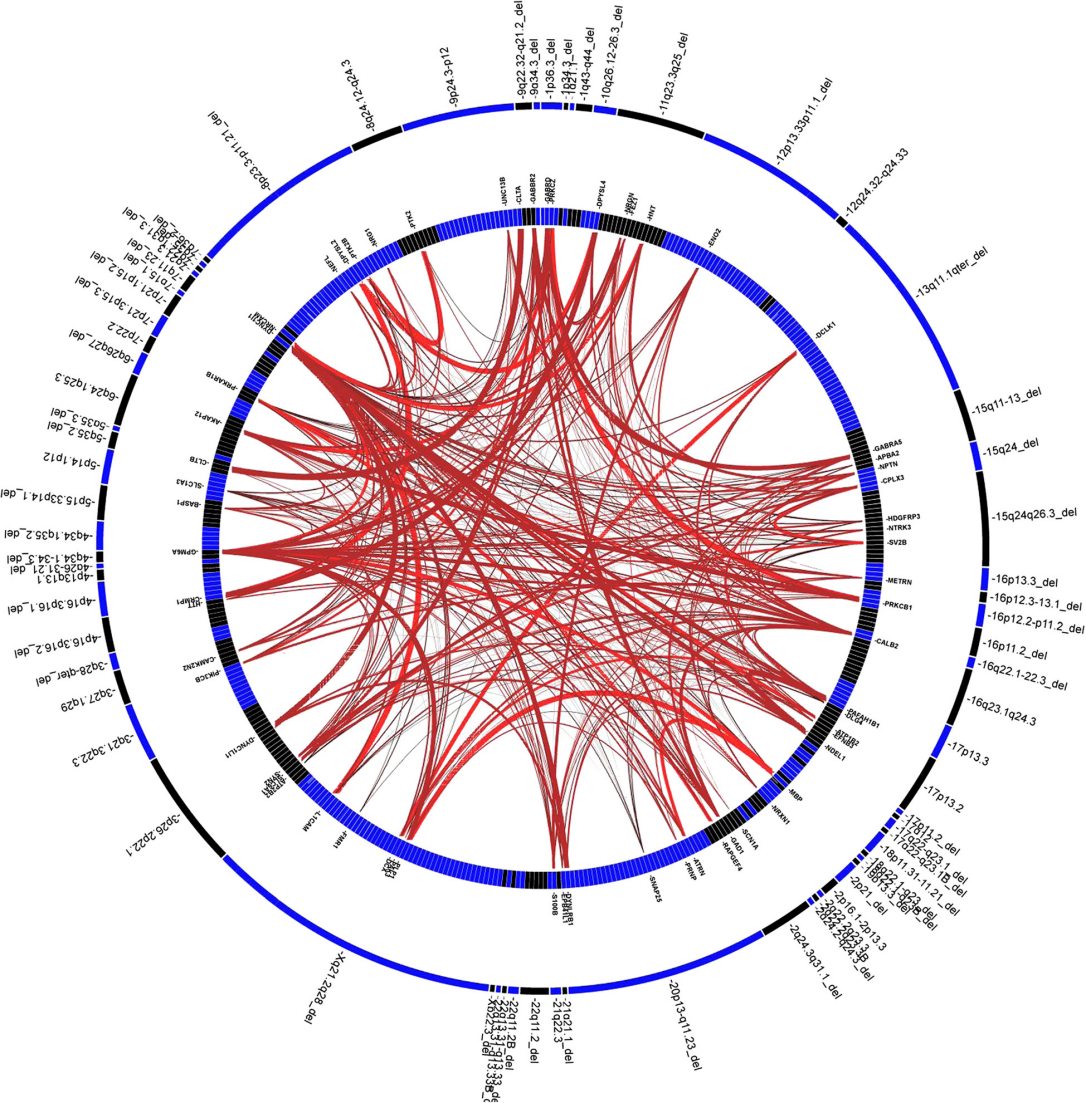
**Functional connections between genes at the 73 critical regions inferred by GRAIL.** The double circle plots depict the gene relationships across the abnormal regions by GRAIL based on data source PubMed Text. Outer circle represents abnormal regions, of which the G-band regions with '_del' are denoted deletions and the other regions are duplications. The inner circle stands for genes located in the corresponding regions in the outer circle, and for clear visualization only significant genes (p < 0.001) and their connections are shown. The lines represent pair-wise gene connections, with the thickness of the lines indicating the degree functional similarity.

To test the hypothesis that there may be functional interrelations of the BEGs across critical regions and identify possible candidate genes, a statistical method GRAIL was applied to assess the degree of relatedness of the listed 461 BEGs [21]. A text-based similarity metric derived from PubMed literature was used to assess the relatedness between genes across the 73 regions. A subset of 108 genes were implicated inter-connection (*ptext* < 0.01) (Table 1), and the words that strongly linked the significant genes in each region were defined as keywords. The top keywords are ‘neuron’, ‘brain’, ‘synaptic’ ‘gaba’ and ‘kinase’, which indicate the common biological functions of these genes. In addition to PubMed literature, other two gene relatedness metrics – Gene Ontology (GO) annotations and gene expression patterns across multiple human tissues were also used. A total of 45 genes obtained *pannotation* < 0.01 with GO similarity and a subset of 317 genes achieved *pexpression* < 0.01 with expression similarity. The results from these three data sources showed that 27 genes were common to all three functional data sources and 74 genes were shared in two functional data sources (Additional file 6: Figure S2). Interestingly, among these candidate genes, *CRMP1*, *PAFAH1B1* and *PDE4D* are spindle related genes, implying that the proper centrosome function is critical for brain development. The top five terms which show strong functional enrichments were neurological process, transmission of nerve pulse, synaptic transmission, cell junction and central nervous system neuron axonogenesis (p < 0.001). The functional connections among implicated genes in the 73 regions were shown in Figure 5 and Additional file 6: Figures S3 and S4.

**Table 1:** List of candidate genes identified by GRAIL

**G-band**	**Candidate gene**	**G-band**	**Candidate gene**	**G-band**	**Candidate gene**	**G-band**	**Candidate gene**
1p36.3	PRKCZ	5q35.2	CLTB	11q23.3q25	HNT	17p13.2	ATP1B2
1p36.3	GABRD	5q35.3	MAPK9	11q23.3q25	NRGN	17p13.2	EFNB3
1q43-q44	AKT3	6q24.1q25.3	AKAP12	11q23.3q25	JAM3	17p13.3	PAFAH1B1
2p21	NRXN1	6q26q27	PDE10A	12p13.33p11.1	KRAS	18q22.1-q23	CBLN2
2q22.2q23.3B	KIF5C	6q26q27	RPS6KA2	12p13.33p11.1	PTPRO	18q22.1-q23	MBP
2q24.3q31.1	STK39	7p22.2	PRKAR1B	12p13.33p11.1	ENO2	20p13-q11.23	DYNLRB1
2q24.3q31.1	GAD1	7q21.3	DYNC1I1	13q11.1qter	DCLK1	20p13-q11.23	PTPRA
2q24.3q31.1	RAPGEF4	7q22.1q31.3	NRCAM	13q11.1qter	MYCBP2	20p13-q11.23	EPB41L1
2q24.3q31.1	SCN1A	7q35	CNTNAP2	13q11.1qter	ITM2B	20p13-q11.23	SNTA1
3p26.2p22.1	DYNC1LI1	7q36.2	DPP6	13q11.1qter	RAP2A	20p13-q11.23	CHGB
3p26.2p22.1	MOBP	8p23.3-p11.21	NEFL	13q11.1qter	SACS	20p13-q11.23	PAK7
3p26.2p22.1	ATP2B2	8p23.3-p11.21	NEFM	13q11.1qter	SLITRK5	20p13-q11.23	SNPH
3p26.2p22.1	SYN2	8p23.3-p11.21	NRG1	13q11.1qter	CLDN10	20p13-q11.23	ATRN
3p26.2p22.1	SLC6A1	8p23.3-p11.21	STMN4	15q11-13	APBA2	20p13-q11.23	PRNP
3q21.3q22.3	PIK3CB	8p23.3-p11.21	DPYSL2	15q11-13	TJP1	20p13-q11.23	SNAP25
3q27.1q29	CAMK2N2	8p23.3-p11.21	PTK2B	15q11-13	GABRA5	21q22.3	S100B
3q27.1q29	SST	8q24.12-q24.3	PTK2	15q24	CPLX3	22q11.2	CLDN5
3q28-qter	APOD	9p24.3-p12	SLC1A1	15q24	NPTN	22q11.2	SEPT5
4p16.3p16.1	CRMP1	9p24.3-p12	DCTN3	15q24q26.3	SV2B	22q13.31-q13.33	MLC1
4p16.3p16.1	WFS1	9p24.3-p12	CLTA	15q24q26.3	NTRK3	Xq21.2q28	L1CAM
4p16.3p16.2	HTT	9p24.3-p12	PTPRD	15q24q26.3	CHRNA3	Xq21.2q28	PAK3
4q34.1-34.3	GPM6A	9p24.3-p12	UNC13B	15q24q26.3	HDGFRP3	Xq21.2q28	DCX
4q34.1-34.3	SCRG1	9q22.32-q21.2	GABBR2	16p12.2-p11.2	PRKCB1	Xq21.2q28	ATP6AP1
5p14.1p12	SLC1A3	10q26.12-26.3	DPYSL4	16p13.3	METRN	Xq21.2q28	PCDH19
5p15.33p14.1	MYO10	11q23.3q25	SORL1	16q22.1-22.3	CALB2	Xq21.2q28	PLXNB3
5p15.33p14.1	BASP1	11q23.3q25	SCN3B	17p13.2	DLG4	Xq21.2q28	FMR1
5q35.2	SNCB	11q23.3q25	FEZ1	17p13.2	NDEL1	Xq21.2q28	PLP1

### Gene network and pathway analysis

**Figure 6:**
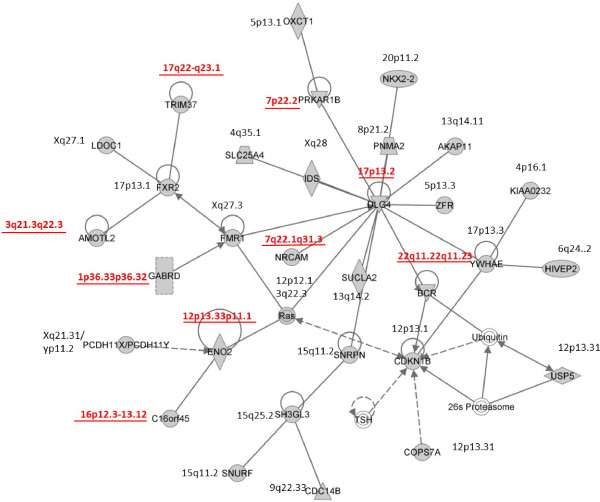
**The primary functional network of BEGs identified by the ingenuity pathway analysis.** Nodes represent genes and a solid line means a direct interaction between the two genes and a dotted line stands for an indirect relationship. Shaded nodes represent genes within the abnormal regions (genes denoted with underlined G-band position are in the candidate gene list of Table 1)

To understand the potential functional interactions of the 461 BEGs, Ingenuity Pathway Analysis (IPA) was performed to construct a network of genes involved in a specific biological process. The highest rated network of genes identified by IPA contains 31 significant genes which function in cell cycle, cell-to-cell signaling and interaction, nervous system development and function (Figure 6). In this network, eight genes including *PRKAR1B, DLG4, NRCAM, GABRD, BCR, AMOTL2, ENO2, C16orf45* were also derived from GRAIL analysis. As shown in Figure 6, *DLG4*, the hub gene in network 1, interacts with 12 genes and has the highest degree (degree = 12, defined as the number of linked genes). IPA also denoted the other top four gene networks related to cellular assembly and organization, cellular function and maintenance and cell morphology. Three of them enriched for genes involved in nervous system development and function (Additional file 6: Figures S5-S8).

## Discussion

Recently, genomic analysis using array-based CGH or SNP chip was recommended as the first-tier genetic test for individuals with ID/DD/MCA and autism because of its high sensitivity and specificity for detecting submicroscopic deletions and duplications [3]. In this study, of the 1354 individuals with ID/DD/MCA/autism analyzed using Agilent’s 44 K and 180 K oligonucleotide arrays, 176 patients (13.0%) were detected with pCNVs, chromosomal structural abnormalities and aneuploidies. This diagnostic yield is in consistent with the reported range of 12%-20% by several studies [5-13,24-26]. Of all the abnormal cases, the largest proportion (37.5%) belongs to recurrent genomic disorders and the other two significant groups are subtelomeric rearrangements (25.6%) and sporadic interstitial abnormalities (18.8%). Recurrent genomic disorders are well recognized to be responsible for syndromic phenotypes. Recently, two case–control studies with large case series further define the phenotypes of recurrent genomic disorders and suggested further effort to develop a genome-wide map for dosage sensitive genes [9,24]. Subtelomeric and interstitial abnormalities showed larger variations in their size distribution and associated phenotypes. Therefore, it will be more difficult to identify the disease-causing genes.

Cytogenomic mapping of disjointed critical regions from the detected genomic imbalances allowed further bioinformatic mining for potential disease-causing genes. Of the 4080 genes sorted from the defined critical regions, about 11.3% (461/4080) of the genes showed brain specific expression by bioinformatic mining. Although the number of BEGs increases with the gene content of genomic regions, the enrichment of genes preferentially expressed in CNS tissues was noted by the gene annotation analysis. This observation suggested that the functional interactions rather than the number of the BEGs are more likely related to the ID phenotype. From the 461 BEGs, a subset of genes was identified with high relatedness through a statistical text mining approach. This enables us to recognize the biological connections across the disease-causing regions. In this subset of highly related genes, five genes have been established as causative genes for well-known mental retardation syndromes. These are *GABRD* for 1p36 deletion and duplication syndrome, *NPTN* for 15q24 BP0-BP1 deletion syndrome, *PAFAH1B1* for 17p13.3 deletion and duplication syndrome, *MECP2* for Rett syndrome, and *FMR1* for fragile X syndrome. The identification of the known causative genes for ID/DD demonstrated the validity of this data mining approach.

ID/DD and autism are complex neurodevelopmental disorders. Recent evidence indicated that defects in synaptogenesis and synaptic activities and deregulation of neuronal development and function may contribute to pathogenesis of ID/DD [1]. Many of the candidate genes recovered by GRAIL are involved in neurological system process, synaptic transmission, cell junction and neuron axonogenesis. Part of the cross-loci interactions of gene ontology from detected deletions and duplications have been presented previously [27]. For example, the *DLG4* gene, a member of synaptic molecules, is a major functional bridge in coupling the N-methyl-D-aspartate receptor to pathways that control bidirectional synaptic plasticity and learning. The phenotypes of *DLG4* knockout mouse have been related to nervous system abnormalities [28]. The *NRXN1* gene which interacts with its postsynaptic binding partner at the synapse has been associated with neurological diseases [29,30]. It is also noted that some of the BEGs did not show significant p-values (e.g, *STS* gene in Xp22.3 has a p-value of 0.83 when text is used). Actually, this exploratory bioinformatic mining was performed on a set of 4,080 genes, about one fifth of the estimated over 20,000 human genes. The inclusion of more abnormal cases and an increase in gene coverage will lead to more comprehensive mining of functional interactions and networks of candidate genes.

The pathway analyses by IPA also found that the BEGs are enriched in neurological disease and endocrine system disorders. The *DLG4*, *FMR1*, *FXR2* and *YWHAE* genes with at least four neighbors (degree > = 4) in Network 1 are all related to neural disorders. The *DLG4* gene is a homologue of a known disease-causing gene *DLG1*. The *FMR1*, *FXR2* and *YWHAE* genes have been reported in ID related studies. Moreover, *SNRPN*, one of the deleted genes for Prader-Willi syndrome, is also directly connected to the *DLG4* gene. This network of genes showed strong functional homogeneity and should be considered a significant network underlying the ID/DD mechanism. Network 2 can be approximately divided into four sub networks connected by the *S100B* gene which is related to neurodegeneration. One of the sub networks involved in cellular assembly and organization included spindle related genes such as *CRMP1*, indicating the important role of centrosome genes in neuron development. Another interesting finding is the detection of significant enrichment in the extracellular signal-regulated kinases (ERK) and mitogen-activated protein kinase (MAPK) signaling pathway by IPA. It has been demonstrated that ERK/MAPK is involved in human genomic disorders [31]. A previous bioinformatic analysis on a small case series showed gene prioritization within MAPK and neuroactive ligand-receptor interaction pathways [26]. A study on 1275 autism cases discovered an enrichment of CNVs disrupting functional gene sets involved in cellular proliferation, projection and motility and GTPase/Ras signaling [32]. Our study identified many significant genes and function pathways overlapped with those previous studies. Further experimental analysis to connect the functional genomics and molecular pathways is required for a better understanding of human brain and nervous system development [33].

## Conclusions

In summary, we performed oligonucleotide aCGH to identify a spectrum of cytogenomic abnormalities including genomic disorders, subtelomeric rearrangements, interstitial imbalances, and chromosomal structural and numerical abnormalities. Further cytogenomic mapping and bioinformatic mining identified a set of candidate BEGs and reveal the underlying functional interrelations and networks likely contributing to the ID/DD phenotypes. The identification of cross-region interrelations of the BEGS could provide clues of related ID phenotypes and possible modifying effect for phenotype variations for better disease classification. The finding of five major networks suggested complex polygenic and multipathway models underlying haploinsufficiency and triple-sensitive regulation of mental development. However, this bioinformatic mining cannot differentiate the driving genetic factors from the modifying effects for the ID phenotypes. Further experimental analysis on animal or cellular models to characterize this complex genetic regulation is needed.

## Methods

### Subjects

The Yale Laboratory of Molecular Cytogenetics and Genomics is CLIA-approved and provides diagnostic services for pediatric patients with indications of ID, DD, MCA, learning disability, speech delay and autism. From 2006 to 2011, aCGH analyses were performed on 1354 pediatric patients using two array platforms: Agilent 44 K oligonucleotide array on 718 patients and Agilent 180 K oligonucleotide array on 636 patients. For cytogenomic mapping and bioinformatic mining of potential candidate genes from the abnormal findings, all patient information had been de-identified. There were no additional pre-study requirements on the patient's specimen and clinical indications and there were no post-study interaction and intervention with the patients. This project was categorized as a chart review retrospective study and deemed exemption from Institutional Review Boards (IRB) approval and waiver of consent based on the policy of Yale University IRB.

### Oligonucleotide array comparative genomic hybridization (aCGH)

Genomic DNA was extracted from peripheral blood lymphocytes using the Gentra Puregene Kit (Qiagen, Valencia, CA). The DNA concentration was measured using a NanoDrop spectrophotometer (Thermo Fisher Scientific, Inc., Waltham, MA) and high molecular weight DNA was verified by agarose gel electrophoresis. For each sample, 2–3 ug of genomic DNA was used following the manufacturer’s protocol for the Agilent Human Genome CGH microarray 44 K kit (containing 44,913 60-mer oligonucleotides) and 180 K kit (177,873 60-mer oligonucleotides) (Agilent Technologies, Inc., Santa Clara, CA). This aCGH procedure can achieve 99% sensitivity and 99% specificity using a sliding window of five to seven contiguous oligonucleotides, indicating an analytical resolution of 300–500 kilobase (Kb) for the 44 K platform and 100–150 Kb for the 180 K platform [4]. The base pair designations of 44 K and 180 K aCGH were based on the May 2004 Assembly (NCBI35/hg17) and the March 2006 Assembly (NCBI36/hg18) of the UCSC Human Genome browser (http://genome.ucsc.edu/), respectively.

The genomic imbalances detected by aCGH were classified as (1) chromosome abnormalities, (2) pathogenic CNV (pCNV), (3) variant of uncertain clinical significance (VOUS) and (4) benign CNV (bCNV) [34]. As previously described, large chromosome abnormalities (>5 Mb) were confirmed by high resolution karyotyping on metaphases prepared from cultured peripheral blood lymphocytes using the laboratory’s standardized protocols; small imbalances were confirmed by FISH using available commercial probes or targeted BAC probes [4]. Raw data of all detected chromosomal imbalances were loaded onto the Nexus5 Software (BioDiscovery, Los Angles, California, USA) to evaluate the genome-wide distribution and relative frequency of chromosomal and genomic abnormalities.

### Fluorescence in situ hybridization (FISH)

To confirm the subtelomeric chromosome rearrangements and common genomic disorders detected by aCGH, FISH using selected ToTelVysion probes and locus-specific probes (Vysis/Abbott, Abbott Park, IL) as well as home brew targeted BAC probes was performed as previously described [4].

### Cytogenomic mapping and bioinformatic analysis

A two-step cytogenomic mapping procedure was used to define critical regions from detected genomic abnormalities. First, segments overlapping with known bCNVs that have been documented at least three times in the Databases of Genomic Variants (DGV) within a pCNV were subtracted. Secondly, pCNVs were compared with other overlapping pCNVs associated with ID/DD from DECIPHER, and the smallest overlapped regions were extracted as critical regions. The gene content within each critical region was obtained based on gene coordinate (hg18) from the UCSC database browser [35]. Subsequent data mining focused on the BEGs from defined critical regions assuming that their functional interactions and networks likely contribute to human mental development.

#### ***Gene functional classification analysis***

The tissue expression analysis was performed using the DAVID Gene Functional Classification Tool with the ‘GNF_U133A_QUARTILE’ option [36]. DAVID would report the tissues in which the gene expresses higher than 3^rd^ quartile of its expression across all tissues. Moreover, the tissue expression patterns were further confirmed by 1,000 runs of permutation tests. For each run, a set of random genes with the same number of genes as that in the critical regions was generated and the number of genes expressed in a specific tissue was recorded.

#### ***Identification and distribution of brain-expressed genes (BEGs)***

A list of BEGs was obtained following the procedures of Raychaudhuri et al. (2009a) [18]. Briefly, a widely used large human normal tissue expression dataset (Human U133A/GNF1H Gene Atlas) [37] was downloaded from the BioGPS website (http://www.biogps.org/) [38]. The downloaded expression data were gcRMA-normalized and averaged across replicates. Genes with expression values < 100 across all the tissues were excluded, since they are considered not expressed. One-tailed Mann–Whitney rank-sum p-values were then obtained to determine whether the genes express higher in the brain or spinal cord (21 tissues) than in the remaining tissue profiles. Genes with *p* < 0.01 were considered as BEGs.

For every defined critical region, a set of 10,000 random genomic loci was produced, each of which comprised of the same number of genes as in the tested critical region, and permutation of the random gene sets was conducted. For a specific region *R*, 10,000 consecutive regions of *N* genes (where *N* is the number of genes in *R*) were sampled from the set of the genes profiled on Human U133A/GNF1H platforms. The number of BEGs in the random gene set was recorded and then compared with that in true abnormal region *R* by boxplots.

#### ***Identification of candidate genes by gene interaction and networking***

To examine the interrelations among the listed BEGs from different critical regions, GRAIL was used to assess the functional similarities by using PubMed abstracts, gene ontology terms or expression patterns in normal human tissues [18]. All critical regions containing BEGs were submitted as both seed and query regions and gene relationships among the abnormal regions were visualized by tools provided on the website (http://www.broadinstitute.org/mpg/grail/ ). The listed BEGs were also imported into the IPA software (Ingenuity Systems Inc; http://www.ingenuity.com/) to identify interactions between the focus genes and other gene objects.

### Web resources

Database of Genomic Variants (DGV): http://projects.tcag.ca/variation/.

DatabasE of Chromosomal Imbalance and Phenotype in Humans using Ensembl Resources (DECIPHER): http://decipher.sanger.ac.uk/.

BioGPS website (http://www.biogps.org/).

Gene Relationships Across Implicated Loci (http://www.broadinstitute.org/mpg/grail/.

Ingenuity Pathways Analysis (Ingenuity Systems Inc., RedwoodCity, California, USA; http://www.ingenuity.com/).

## Abbreviations

aCGH: Array comparative genomic hybridization; BEGs: Brain-expressed genes; CNV: Copy number variant (pCNV, pathogenic; bCNV: Benign; VOUS: Variant of uncertain clinical significance); DD: Developmental disability; ID: Intellectual disability; LCRs: Low copy number repeats; MCA: Multiple congenital anomalies.

## Competing interests

All authors declare that they have no competing interests.

## Authors’ contributions

FX carried out the 180 K aCGH and organized cytogenomic findings, LL performed most bioinformatic analysis. FX and LL drafted the manuscript. VPS and PGG performed the IPA gene networking analysis. BX carried out the 44 K aCGH analysis, HZ supervised the bioinformatic and statistical analyses. HZ, PGG and PL participated in the design and coordinate of this study. All authors read and approved the final version of the manuscript.

## Supplementary Material

Additional file 1: Table S1 Detected recurrent genomic disorders. Click here for file

Additional file 2: Table S2 Subtelomeric chromosomal and genomic rearrangements. Click here for file

Additional file 3: Table S3 Sporadic interstitial chromosomal and genomic imbalances. Click here for file

Additional file 4: Table S4 Chromosome structural abnormalities. Click here for file

Additional file 5: Table S5 Disjointed critical regions defined by cytogenomic mapping. Click here for file

Additional file 6: Figure S1 The distribution for the number of genes in a specific tissue. Arrow points to number of genes from abnormal regions. **Figure S2.** The number of candidate genes overlapping between the three data sources. **Figure S3.** Functional connections between genes at the 73 critical regions inferred by GRAIL based on Gene Ontology (significant genes with p < 0.01). **Figure S4.** Functional connections between genes at the 73 critical regions inferred by GRAIL based Human Expression Alta (significant genes with p < 0.0000000001). **Figure S5.** The secondary functional network of BEGs identified by the Ingenuity pathway analysis (genes denoted with red colored G-band position are in the candidate gene list of Table 1). **Figure S6.** The third functional network of BEGs identified by the Ingenuity pathway analysis (genes denoted with red colored G-band position are in the candidate gene list of Table 1). **Figure S7.** The fourth functional network of BEGs identified by the Ingenuity pathway analysis (genes denoted with red colored G-band position are in the candidate gene list of Table 1). **Figure S8.** The fifth functional network of BEGs identified by the Ingenuity pathway analysis (genes denoted with underlined G-band position are in the candidate gene list of Table 1). Click here for file

Additional file 7: Table S6 List of brain expressed genes. Click here for file
