# Supplementary material for: Cytogenomic mapping and bioinformatic mining reveal interacting brain expressed genes for intellectual disability
Source: Mol Cytogenet. 2014 Jan 10;7:4. doi: 10.1186/1755-8166-7-4 (PMC3905969; doi:10.1186/1755-8166-7-4)
Supplement: Additional file 6: Figure S1 — The distribution for the number of genes in a specific tissue. Arrow points to number of genes from abnormal regions. Figure S2. The number of candidate genes overlapping between the three data sources. Figure S3. Functional connections between genes at the 73 critical regions inferred by GRAIL based on Gene Ontology (significant genes with p < 0.01). Figure S4. Functional connections between genes at the 73 critical regions inferred by GRAIL based Human Expression Alta (significant genes with p < 0.0000000001). Figure S5. The secondary functional network of BEGs identified by the Ingenuity pathway analysis (genes denoted with red colored G-band position are in the candidate gene list of Table 1). Figure S6. The third functional network of BEGs identified by the Ingenuity pathway analysis (genes denoted with red colored G-band position are in the candidate gene list of Table 1). Figure S7. The fourth functional network of BEGs identified by the Ingenuity pathway analysis (genes denoted with red colored G-band position are in the candidate gene list of Table 1). Figure S8. The fifth functional network of BEGs identified by the Ingenuity pathway analysis (genes denoted with underlined G-band position are in the candidate gene list of Table 1). [file 1755-8166-7-4-S6.ppt]

## Slide 1
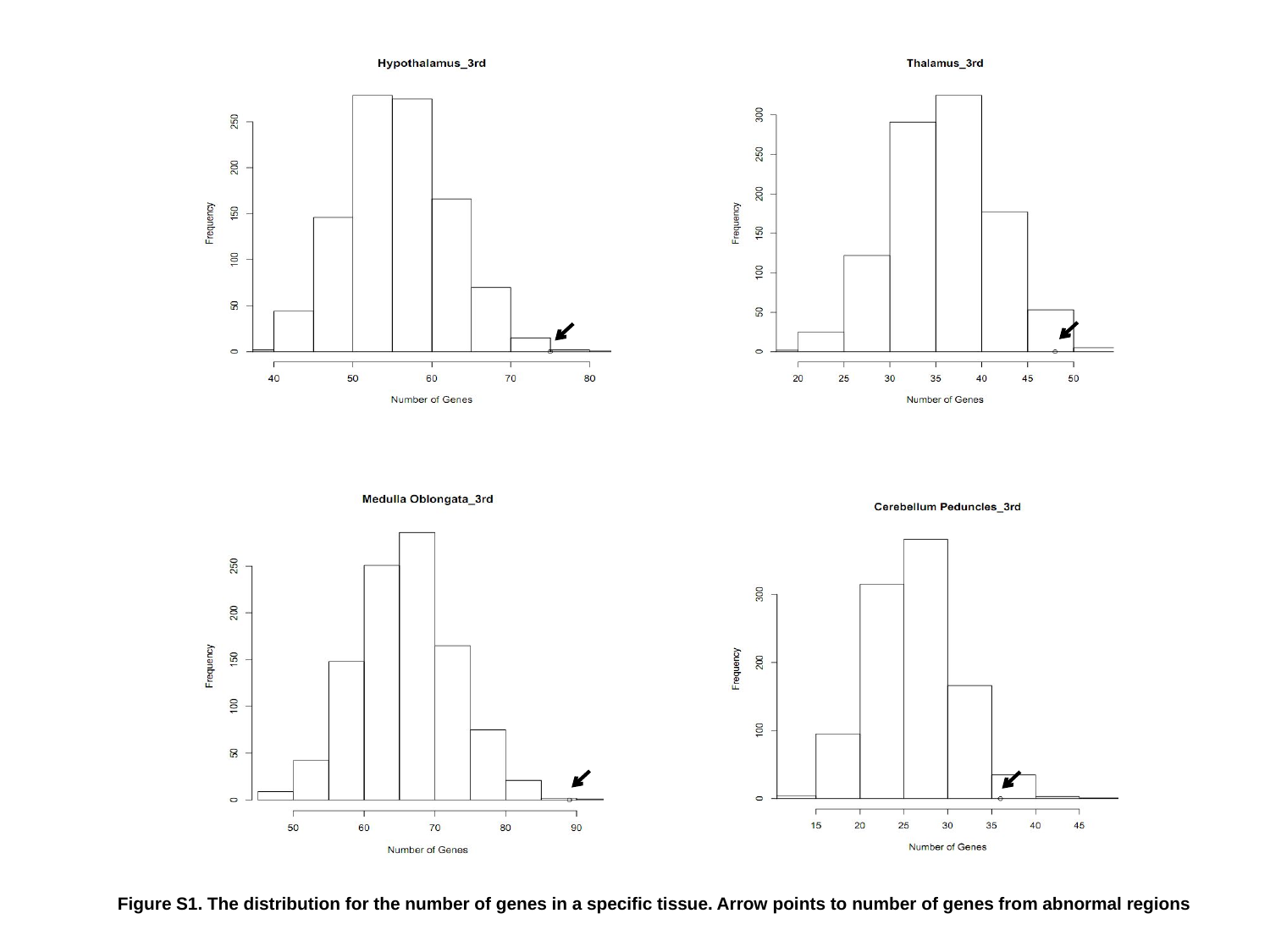

Figure S1. The distribution for the number of genes in a specific tissue. Arrow points to number of genes from abnormal regions

## Slide 2
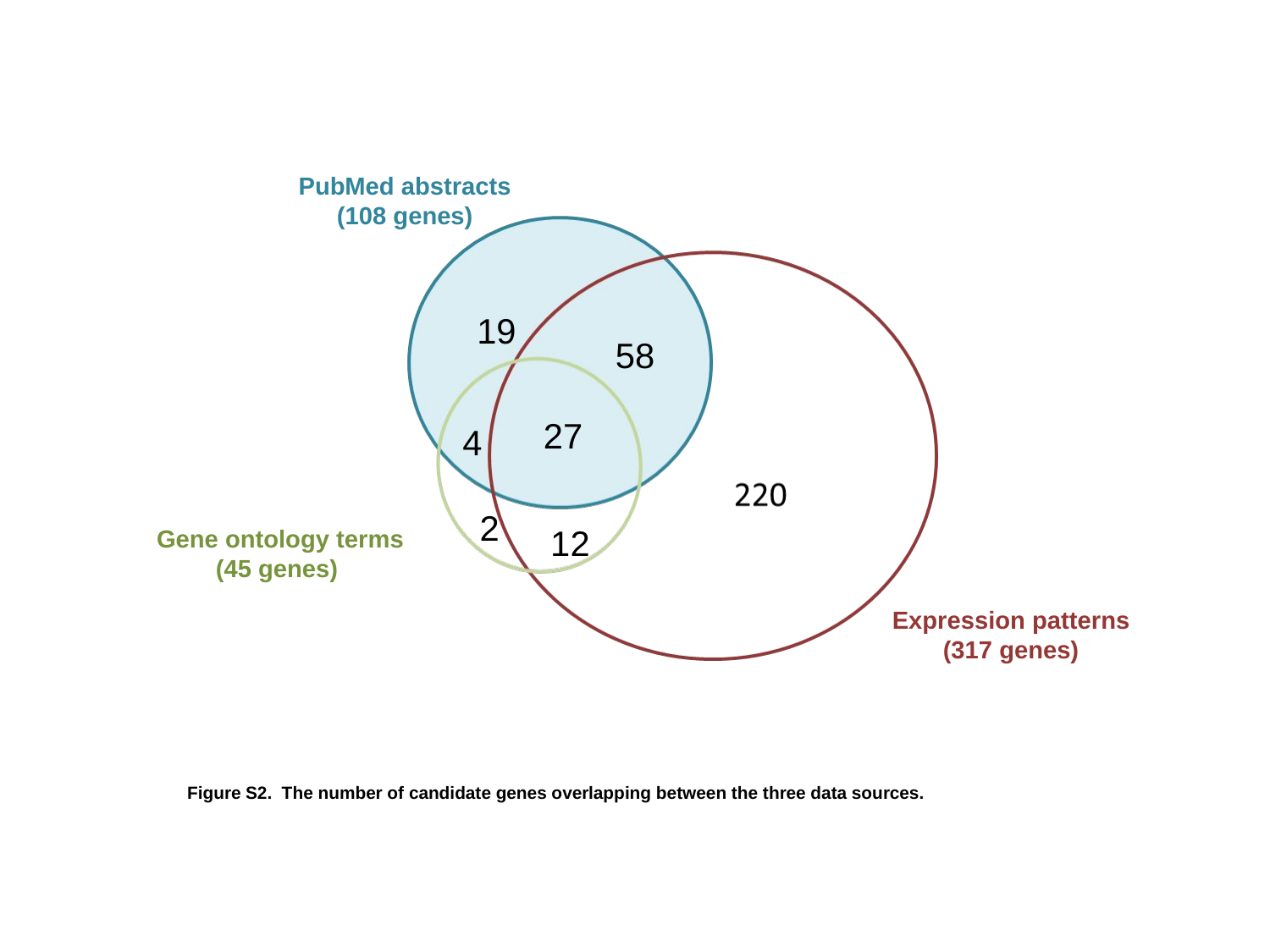

PubMed abstracts
(108 genes)
19
58
27
4
2
12
Gene ontology terms
(45 genes)
Expression patterns
(317 genes)
Figure S2. The number of candidate genes overlapping between the three data sources.

## Slide 3
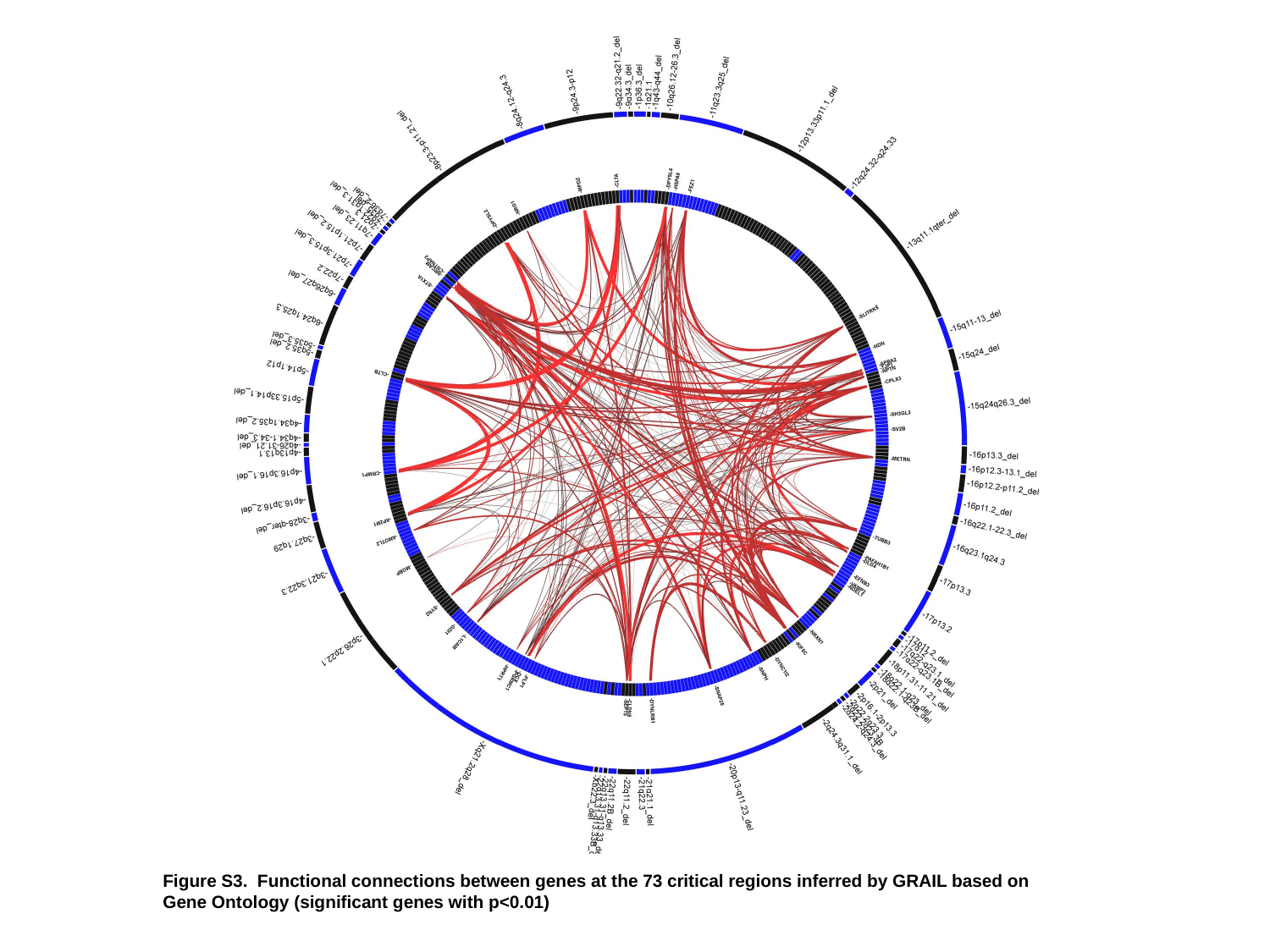

Figure S3. Functional connections between genes at the 73 critical regions inferred by GRAIL based on
Gene Ontology (significant genes with p<0.01)

## Slide 4
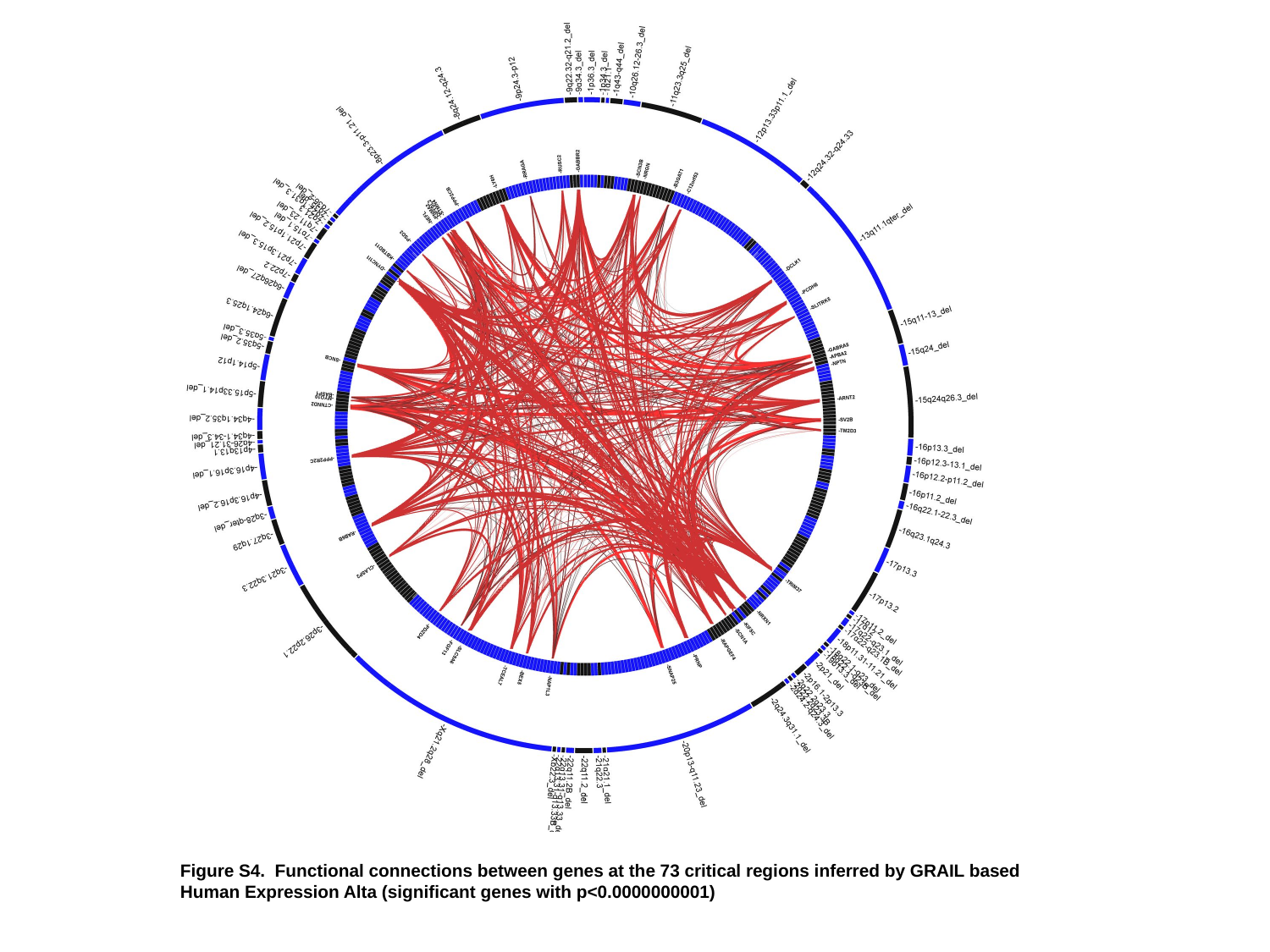

Figure S4. Functional connections between genes at the 73 critical regions inferred by GRAIL based
Human Expression Alta (significant genes with p<0.0000000001)

## Slide 5
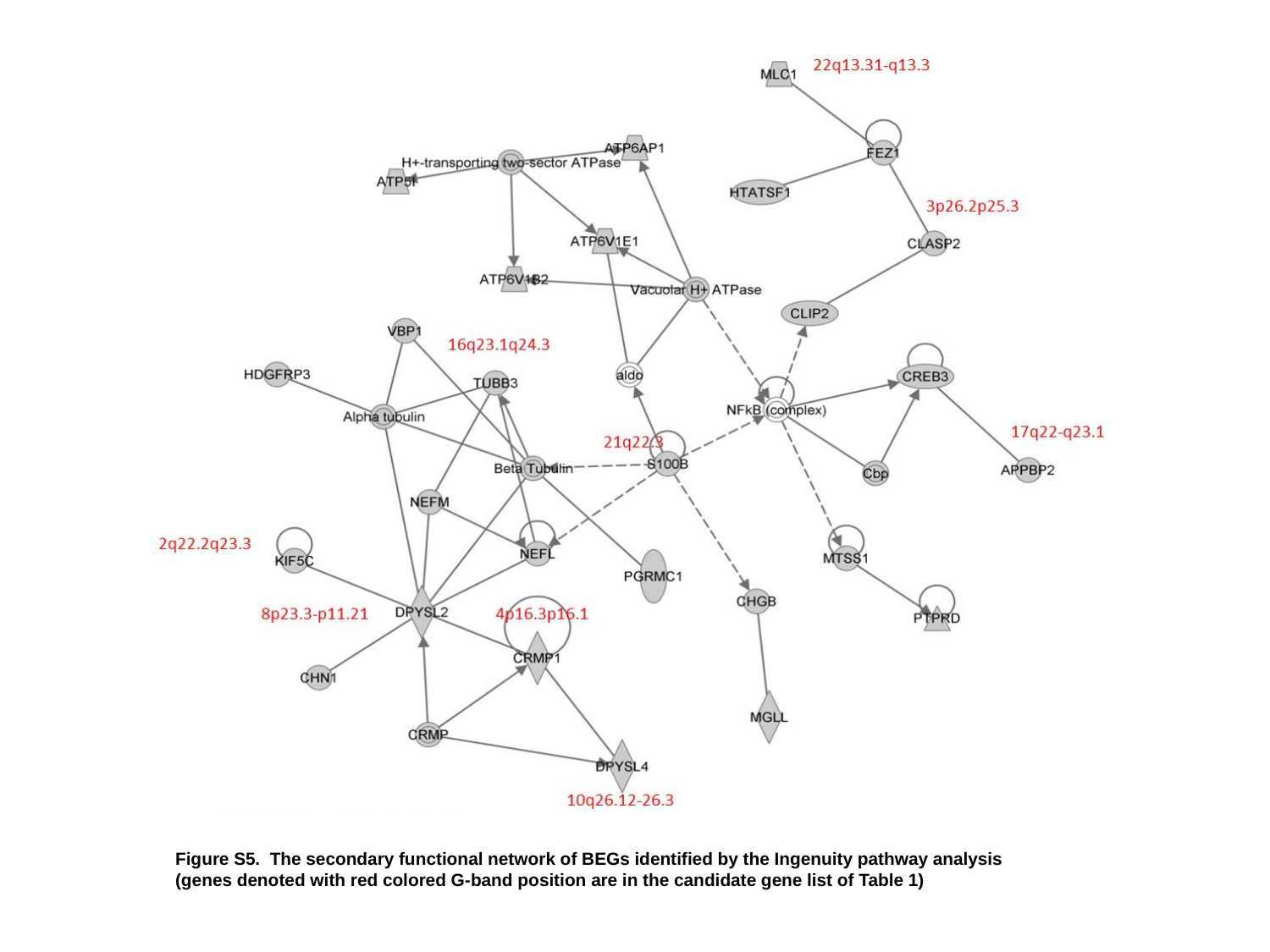

Figure S5. The secondary functional network of BEGs identified by the Ingenuity pathway analysis
(genes denoted with red colored G-band position are in the candidate gene list of Table 1)

## Slide 6
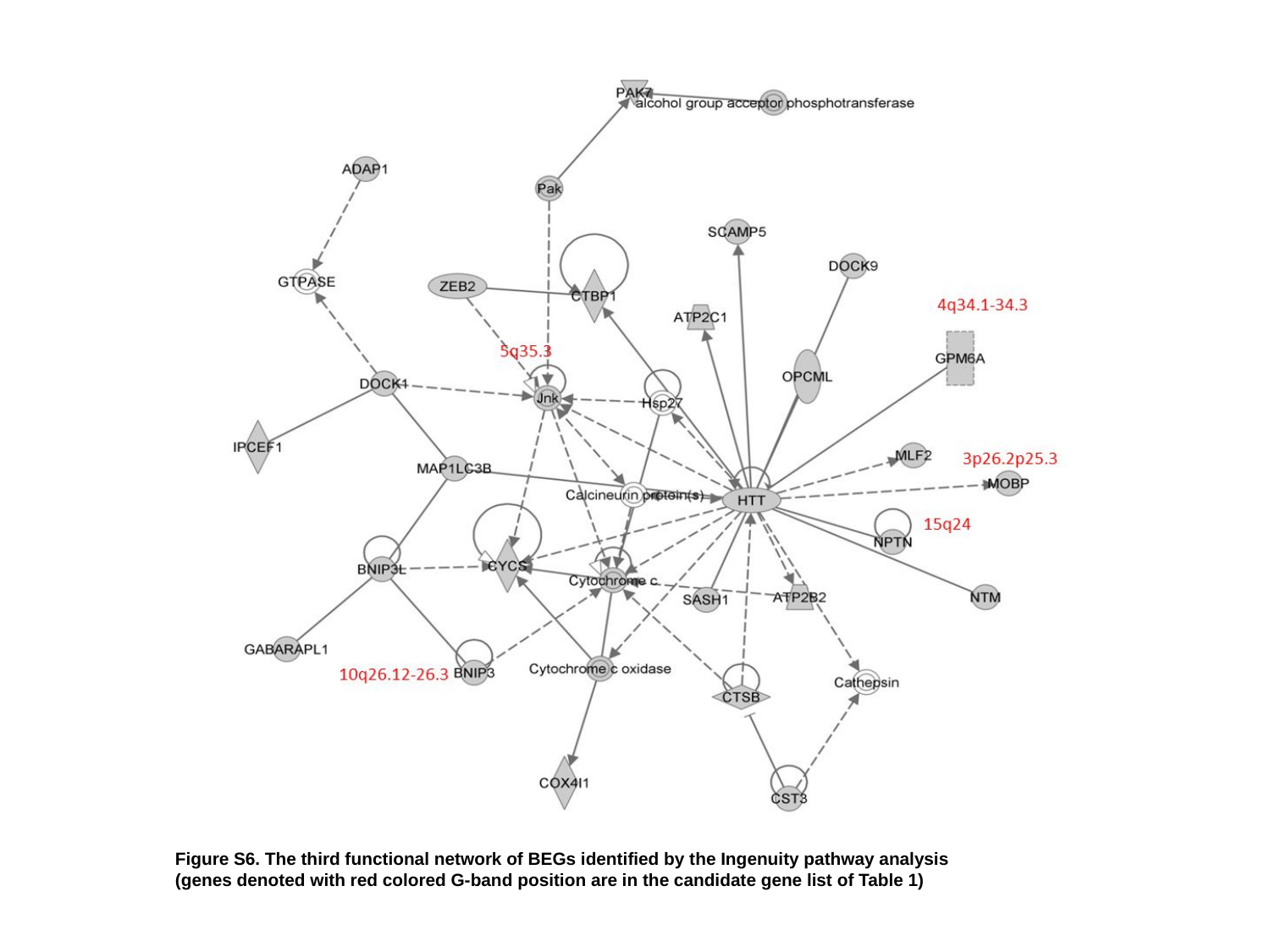

Figure S6. The third functional network of BEGs identified by the Ingenuity pathway analysis
(genes denoted with red colored G-band position are in the candidate gene list of Table 1)

## Slide 7
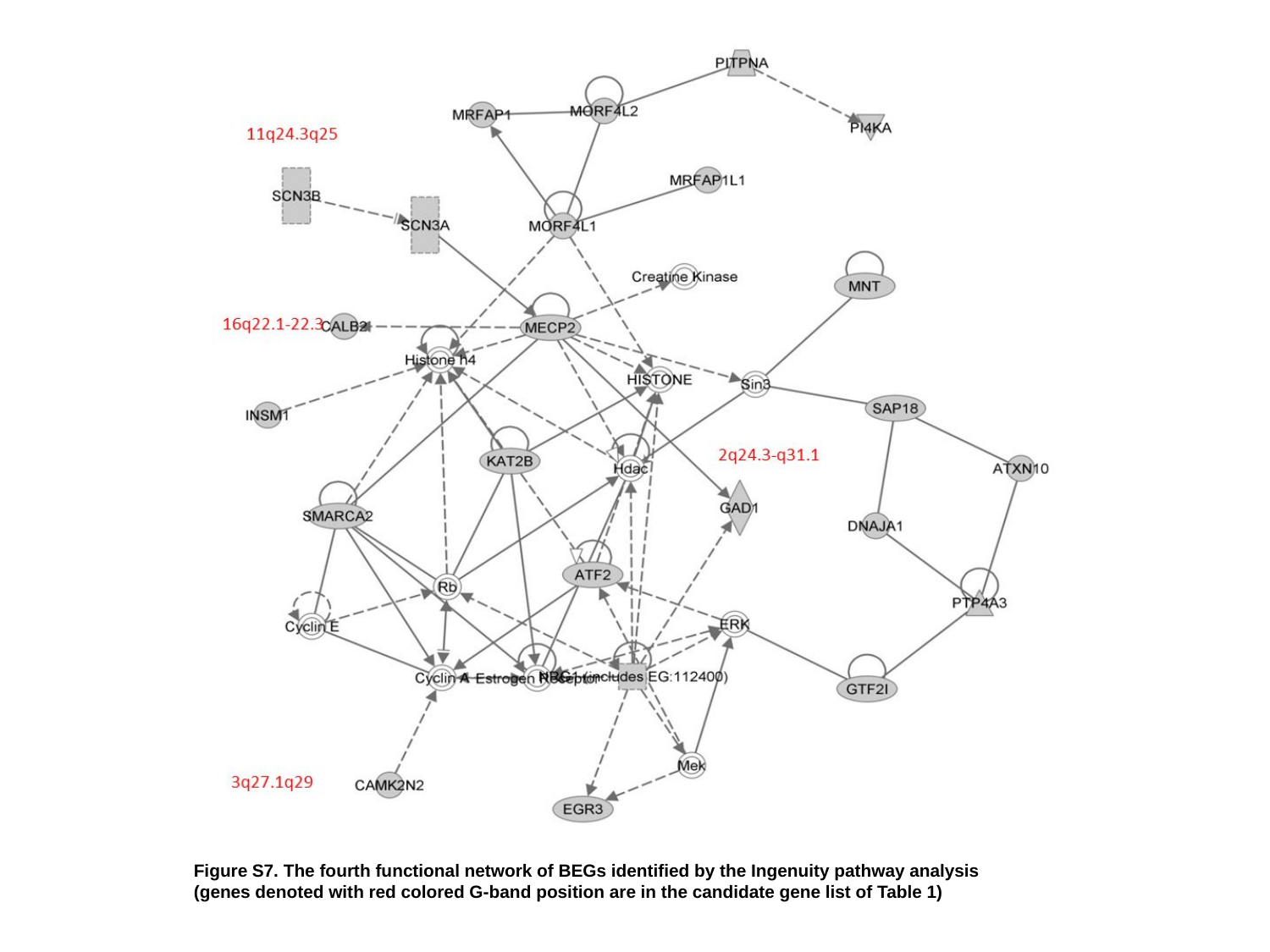

Figure S7. The fourth functional network of BEGs identified by the Ingenuity pathway analysis
(genes denoted with red colored G-band position are in the candidate gene list of Table 1)

## Slide 8
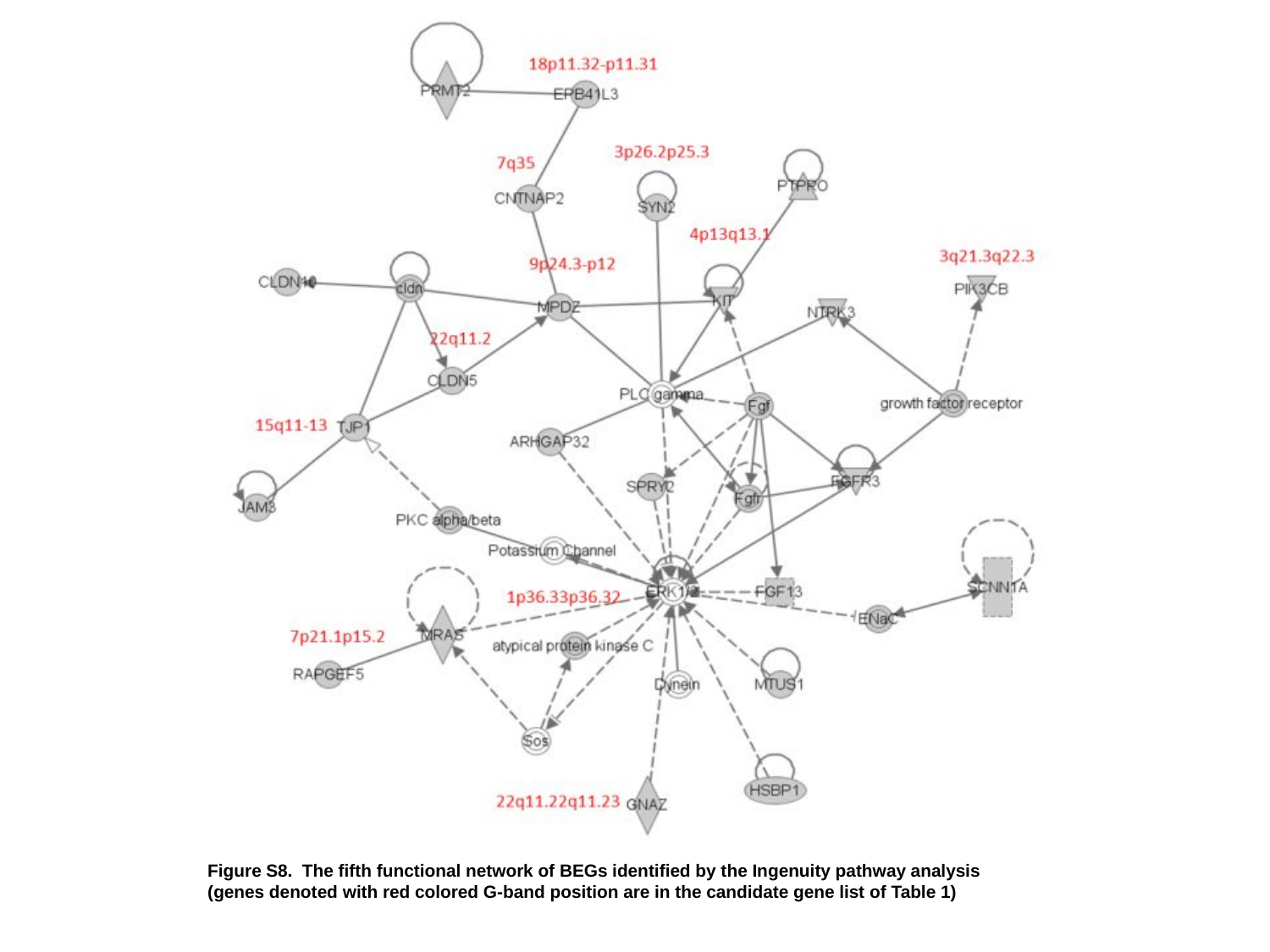

Figure S8. The fifth functional network of BEGs identified by the Ingenuity pathway analysis
(genes denoted with red colored G-band position are in the candidate gene list of Table 1)
